# Unique patterns of healthcare utilization following the opening of the Samson Assuta Ashdod University Hospital

**DOI:** 10.1038/s41598-023-41758-2

**Published:** 2023-09-12

**Authors:** Noa Dror Lavy, Royi Barnea, Erela Rotlevi, Tzahit Simon-Tuval

**Affiliations:** 1 Department of Health Policy and Management, Guilford Glazer Faculty of Business and Management and Faculty of Health Sciences, Ben-Gurion University of the Negev, P. O. Box 653, 8410501 Beer-Sheva, Israel; 2 Maccabi Health Service, Netanya, Israel; 3 Assuta Health Services Research Institute, Assuta Medical Centers, Tel-Aviv, Israel; 4 School of Health Systems Management, Netanya Academic College, Netanya, Israel

**Keywords:** Health care economics, Health policy, Health services

## Abstract

Our aim was to examine the influence of the market entry of Samson Assuta Ashdod University Hospital on community and hospital-based healthcare utilization (HCU). A retrospective study was conducted among Maccabi Health Services enrollees in the regions of Ashdod (n = 94,575) and Netanya (control group, n = 80,200) before and after this market entry. Based on difference-in-differences framework, we examined the change in HCU of Ashdod region’s enrollees compared to the control group and following the market entry using multivariable generalized estimating equations models. Our results revealed that, as hypothesized, after the market entry and compared to the control group, there was a 4% increase in specialists visits not requiring referral (RR = 1.04, 95% CI 1.03–1.06, *p* < 0.001), a 4% increase in MRI and CT scans (RR = 1.04, 95% CI 1.01–1.08, *p* = 0.022), and a 33% increase in emergency room visits (RR = 1.33, 95% CI 1.29–1.38, *p* < 0.001). Unexpectedly, no changes were observed in the number of hospital admissions (RR = 1.05, 95% CI 0.97–1.14, *p* = 0.250), and hospitalization days (RR = 0.99, 95% CI 0.94–1.04, *p* = 0.668). Moreover, and unexpectedly, there was a 1% decrease in primary care physician visits (RR = 0.99, 95% CI 0.98–1.00, *p* = 0.002), a 11% decrease in specialists visits requiring a referral (RR = 0.89, 95% CI 0.86–0.91, *p* < 0.001), and a 42% decrease in elective surgeries (RR = 0.58, 95% CI 0.55–0.60, *p* < 0.001). We conclude that this market entry was not translated to an increase in utilization of all services. The unique model of maintaining the continuity of care that was adopted by the hospital and patients’ loyalty may led to the unique inter-relationship between the hospital and community care.

## Introduction

The healthcare system exhibits asymmetric information that may lead to supplier-induced demand^1, 2^. Many studies have investigated this phenomenon in a wide range of healthcare services including hospitalizations, scanning tests [magnetic resonance imaging (MRI) and computerized tomography (CT)], physician visits, medication, medical innovation (new preventive technologies, diagnostic innovation), and outpatient surgical procedures^3–8^. According to Roemer's law^9^, excess hospital beds lead to overutilization of hospital services, and the observed demand outpaces the population’s actual need for services. An Australian study found that opening of an emergency department within the region led to an 18% increase in the total volume of emergency room visits, while the local population growth was only 3%, suggesting the service either provided an unmet need or overutilization^10^. However, empirical evidence for Roemer's law is not unequivocal^4, 11^. Thus, the effect of hospital market entry on service volume still needs to be examined, along with its influence on pre-existing healthcare providers^10, 12^.

Payment schemes incentivize healthcare provider’s activity as well, and thus, also influence efficiency, quality, availability, equality of care, and healthcare utilization (HCU). Each payment method has its strengths and weaknesses and their influence on these aspects needs to be carefully considered and continuously monitored. Many studies have been conducted to examine this effect^13^. Each payment method generates incentives and affects provider’s behavior and may influence the volume of healthcare services and health spending^13^. Payment schemes that incentivize providers to increase activity include fee-for-service (FFS)^7, 14–17^, diagnosed-related group (DRG)^18^, and per diem^19^. A payment scheme that incentivizes providers to decrease activity is global budget since it is unrelated to the volume of clinical activity^20^ and may lead to reduced quality of care and accessibility^13^.

Since enactment of the Israeli National Health Insurance Law in 1995, Israeli residents have had universal health insurance coverage. Citizens are free to enroll in one of four nonprofit health maintenance organizations (HMOs) that are obligated to provide them equal access to a generous benefits package including physician consultations, ambulatory care, hospitalizations, and medications. The HMOs are allowed to apply copayment charges subject to strict governmental authorization^21^. Maccabi Healthcare Services (MHS) is the second largest HMO in Israel, insuring 2.5 million members (30.9% of the total Israeli population) in 2021^22^. Thus, MHS as a single-payer setting enables to provide reliable and comprehensive measures of HCU data for our study population. The Samson Assuta Ashdod University Hospital (henceforth: Assuta Ashdod) of MHS was opened in June 2017 and serves about half a million people, who are residents of the city of Ashdod and the surrounding area and are enrollees of all four HMOs in Israel. Assuta Ashdod applies a new advanced model of public health—“a community that has a hospital”—which provides patient services that address all their medical and social needs. This advanced model requires integrative work with the community, municipality, and welfare representatives to create a therapeutic continuum. Thus, it may change the HCU of Ashdod residents in particular, and of Southern District residents, in general.

The market entry of Assuta Ashdod provided a unique opportunity for a natural experiment to examine whether the hospital entry led to increased utilization of inpatient and outpatient healthcare services, and a shift in the geographical distribution of HCU. This analysis may serve as a model for future analysis of market entry of a tertiary medical center into health systems worldwide and will enhance planning of resource allocation following similar market entries. Thus, it may optimize the distribution of community-based and hospital-based services.

## Methods

### Study design and setting

A retrospective study was conducted among MHS enrollees in the Ashdod and Netanya regions in Israel before and after the market entry of Assuta Ashdod. The hospital was opened in stages, starting in June 2017 with the opening of outpatient clinics, and completion in November 2017 with the opening the emergency department. During the intervening months, various departments and services were gradually opened. Therefore, to avoid biased estimates of HCU, the pre-hospital opening period included seven years prior to June 2017 (June 2010–May 2011, June 2011–May 2012, June 2012–May 2013 June 2013–May 2014, June 2014–May 2015, June 2015–May 2016, June 2016–May 2017), while the post-hospital opening period included the 2 years following December 2017 (January 2018–December 2018, January 2019–December 2019). The study protocol was approved by the MHS Internal Review Board–Helsinki Committee (#MHS-0068-20), and participants’ informed consent was waived by this committee since this was a secondary analysis of a de-identified dataset. All methods were carried out in accordance with relevant guidelines and regulations.

### Study population

The study included all MHS enrollees who were residents of the Ashdod region, which includes the city of Ashdod as well as nearby localities (n = 94,575 before the market entry and n = 84,388 after). Residents of the Netanya region served as control subjects. This region includes the city of Netanya as well as nearby localities (n = 80,200 before the market entry and n = 75,363 after). It was chosen as a control group since its population characteristics are similar, yet no market entry took place. Netanya is located ⁓ 70 km from Ashdod. Several tertiary medical centers provide services to the region residents. Specifically, Laniado hospital is located in the city, and Meir medical Center, Hillel Yaffe Medical Center, Tel Aviv Sourasky Medical Center and Sheba Medical Center are located 20–40 km from Netanya. Although Netanya residents are eligible for receiving services from Assuta Ashdod (as from all providers in Israel), in fact after the market entry of Assuta Ashdod, MHS enrollees in this region were not hospitalized or did not undergo elective surgeries in this medical center. No exclusion criteria were applied. For the comparison of study population characteristics before the market entry, the most recent demographic and clinical characteristics of the study population in each region were extracted. These included: age, sex, morbidities (cardiovascular disease, diabetes, hypertension, kidney disease, and cancer), supplementary health insurance coverage, and socioeconomic status (SES). However, for most of the analyses, the annual levels of these variables were extracted. SES was defined based on the Central Bureau of Statistics classification, ranging from 1 (low SES) to 10 (high SES). This index characterizes geographical units using a combination of demographic composition, schooling and education, standard of living, employment, and benefits of residents^23, 24^.

### Healthcare utilization

All study variables were obtained from the detailed and comprehensive claims data of MHS that document all the services it provides or reimburses. Patient-level HCU data was based on procedure codes and included: primary care visits, specialist care visits requiring referral from a primary care physician (gastroenterology and cardiology), specialist visits not requiring referral from a primary care physician (ophthalmology, orthopedics, gynecology, general surgery, dermatology, otolaryngology), MRI and CT scans, elective surgeries, emergency room visits, and hospitalizations. HCU data were estimated for each of the 9 years of follow-up by type of service and provider geographical location. If the subject was an active member of MHS yet, did not utilize the specific service in a specific year, a zero (rather than a null) value was included in the database. The annual average HCU for each subject was calculated by dividing the sum of quantity (visits, days, scans, surgeries) by the number of years in which the subject was an active MHS member.

### Statistical analysis

Univariable analyses were performed to examine between-group differences in HCU, as well as within-group changes in HCU before and after the market entry of Assuta Ashdod using a Mann–Whitney U test. Similar test was applied to examine the change in HCU of Ashdod region’s residents in providers located inside and outside Ashdod before and after this market entry. We estimated eight multivariable generalized estimating equations (GEE) models, assuming log-link function and negative binomial distribution to examine the effect of the market entry on each service separately following a difference-in-differences specification:

1 $$y_{it} = \beta_{0} + \beta_{1} \cdot post_{it} + \beta_{2} \cdot region_{it} + \gamma \cdot post_{it} \cdot region_{it} + \beta_{3} \cdot t_{i} + \beta_{4} \cdot x_{it1} \ldots + \beta_{13} \cdot x_{it10} + u_{it}$$

where our exponentiated coefficient of interest is of the interaction between region [Ashdod (study group) vs. Netanya (control group)] and post-market entry (vs. pre-market entry), $\gamma$, which reflects the unique change in HCU of Ashdod residents after the hospital market entry. All models were adjusted to age, sex, comorbidity burden, supplementary health insurance coverage, and SES (captured by $\beta_{4} - \beta_{13}$).

Data were analyzed using STATA software (version 17.0, StataCorp, College Station, TX, USA). *p* value < 0.05 determined statistical significance in all analyses.

## Results

Our cohort consisted of 94,575 enrollees of Ashdod region and 80,200 enrollees of Netanya region at baseline (pre-hospital market entry). Table 1 presents a between-group comparison at baseline. The analysis revealed significant differences in all demographic and clinical characteristics, except of prevalence of cardiovascular disease (*p* = 0.083). However, most of the differences are not considerable. Specifically, Ashdod region’s enrollees were younger (32.5 vs. 36.3, *p* < 0.001) and lower proportion were female (50.4 vs. 51.2, *p* < 0.001). In addition, a higher proportion of the study population was covered by supplementary health insurance (87.8% vs. 87.4%, *p* = 0.015), however, SES was lower in Ashdod (4.60 vs. 6.12, *p* < 0.001). Regarding comorbidity burden, compared to Netanya region, lower prevalence was observed among Ashdod enrollees in kidney disease (5.8% vs. 6.7%, *p* < 0.001), and oncology disorders (3.9% vs. 5.0%, *p* < 0.001). On the other hand, a higher prevalence was observed in diabetes (6.9% vs. 6.3%, *p* < 0.001), and hypertension (16.2% vs. 15.0%, *p* < 0.001). Nevertheless, all multivariable models were adjusted to these variables.

**Table 1:** Between-group comparison of study population characteristics pre-market entry.

	Ashdod region (study group)	Netanya region (control group)	*p* value
*n*	94,575	80,200	
Age^a^	32.54 ± 22.96 (29.00, 36.00)	36.28 ± 24.19 (35.00, 40.00)	*p* < 0.001^b^
Female (%)	50.38	51.20	*p* < 0.001^c^
Morbidities (%)			
Cardiovascular disease	4.90	5.08	*p* = 0.083^c^
Diabetics	6.85	6.32	*p* < 0.001^c^
Hypertension	16.18	15.01	*p* < 0.001^c^
Kidney disease	5.75	6.72	*p* < 0.001^c^
Oncology disorders	3.87	5.02	*p* < 0.001^c^
Private health insurance coverage (%)	87.75	87.37	*p* = 0.015^c^
Socioeconomic status^a,d^	4.60 ± 1.70 (5.00, 3.00)	6.12 ± 1.61 (6.00, 2.00)	*p* < 0.001^b^

^a^Values are average ± SD (median, IQR).

^b^Mann–Whitney U test.

^c^χ^2^ test.

^d^Socioeconomic status ranges between 1(the lowest level) and 10 (the highest level), according to Points (Location Intelligence) measurement^23, 24^.

Table 2 presents between-group differences as well as with-group change in HCU following the hospital market entry. Except of the utilization of emergency room visits that were higher at baseline in Ashdod region compared to Netanya region (0.53 vs. 0.40, *p* < 0.001), and the utilization of MRI and CT scans and elective surgeries that was relatively similar (0.08 vs. 0.08, *p* > 0.05; 0.06 vs. 0.06, *p* < 0.001, respectively), a lower utilization of all other services in Ashdod region was observed (Table 2). In addition, the within-group changes in HCU following the hospital market entry revealed that while similar trends were observed in both regions in primary care visits, MRI and CT scans and emergency room visits; opposite trends were observed in the two regions in specialist care visits, hospitalizations, and elective surgeries (Table 2).

**Table 2:** Between-group differences and within-group change in the average annual healthcare utilization.

Type of service	Ashdod region (study group)			Netanya region (control group)		
	Pre-market entry	Post-market entry	*p* value^a^	Pre-market entry	Post-market entry	*p* value^a^
Primary care visits	5.85 ± 5.27 (4.57, 5.83)*	6.27 ± 5.82 (5.00, 7.00)	*p* < 0.001	6.08 ± 5.67 (4.57, 6.33)	6.45 ± 6.33 (5.00, 7.00)	*p* < 0.001
Specialist care visits (not requiring referral from a primary care physician)^b^	2.31 ± 3.00 (1.29, 2.50)**	2.42 ± 3.59 (1.00, 2.50)^#^	*p* < 0.001	2.61 ± 3.27 (1.50, 3.07)	2.55 ± 3.78 (1.00, 3.50)	*p* < 0.001
Specialist care visits (requiring referral from a primary care physician)^c^	0.26 ± 0.73 (0.00, 0.14)**	0.25 ± 0.80 (0.00, 0.00)^##^	*p* < 0.001	0.42 ± 1.42 (0.00, 0.33)	0.43 ± 1.62 (0.00, 0.00)	*p* < 0.001
MRI and CT scans	0.08 ± 0.28 (0.00, 0.00)	0.13 ± 0.45 (0.00, 0.00)^##^	*p* < 0.001	0.08 ± 0.28 (0.00, 0.00)	0.12 ± 0.44 (0.00, 0.00)	*p* < 0.001
Emergency room visits	0.53 ± 2.16 (0.14, 0.50)**	0.71 ± 2.95 (0.00, 0.50)^##^	*p* < 0.001	0.40 ± 0.75 (0.14, 0.50)	0.42 ± 0.94 (0.00, 0.50)	*p* < 0.001
Number of hospital admissions	0.09 ± 0.41 (0.00, 0.00)**	0.09 ± 0.48 (0.00, 0.00)^##^	*p* < 0.001	0.12 ± 0.48 (0.00, 0.00)	0.11 ± 0.56 (0.00, 0.00)	*p* < 0.001
Hospitalization days	0.28 ±1.92 (0.00, 0.00)**	0.27 ± 2.68 (0.00, 0.00)^##^	*p* < 0.001	0.35 ± 2.05 (0.00, 0.00)	0.32 ± 2.56 (0.00, 0.00)	*p* < 0.001
Elective surgeries	0.06 ± 0.21 (0.00, 0.00)**	0.05 ± 0.21 (0.00, 0.00)^##^	*p* < 0.001	0.06 ± 0.18 (0.00, 0.00)	0.08 ± 0.27 (0.00, 0.00)	*p* < 0.001

Values are average ± SD (median, IQR).

^a^Within-group change post-market entry, Mann–Whitney U test.

^b^Ophthalmology, orthopedics, gynecology, general surgery, dermatology, otolaryngology.

^c^Gastroenterology and cardiology.

*Between-group comparison, Mann–Whitney U test pre-market entry *p* < 0.05; ** *p* < 0.001.

^#^Between-group comparison, Mann–Whitney U test post-market entry *p* < 0.05; ## *p* < 0.001.

Table 3 presents the differences in the geographical distribution of HCU of Ashdod region’s enrollees. As hypothesized, in all healthcare services examined (except specialists visits to gastroenterologists and cardiologists), utilization of providers located in Ashdod increased, while outside of Ashdod, it decreased. Specifically, the most pronounced increase in the use of Ashdod providers was in emergency room visits (0.13 vs.0.54, *p* < 0.001), specialist visits not requiring referral (1.89 vs. 2.59, *p* < 0.001), and MRI and CT scans (0.05 vs. 0.11, *p* < 0.001). Unexpectedly, there was a minor decrease in gastroenterology and cardiology specialist visits requiring referral (0.21 vs. 0.20, *p* < 0.001). An opposite trend in HCU was observed in providers located outside of Ashdod. The number of emergency room visits (0.40 vs. 0.16, *p* < 0.001), total hospitalization days (0.28 vs. 0.11, *p* < 0.001), and number of hospital admissions (0.09 vs. 0.03, *p* < 0.001) all decreased following the market entry.

**Table 3:** Within-group change in average annual healthcare utilization of Ashdod region’s enrollees in providers located in Ashdod versus outside of Ashdod pre- and post-market entry.

Type of service	Providers in Ashdod			Providers outside of Ashdod		
	Pre-market entry	Post-market entry	*p* value^a^	Pre-market entry	Post-market entry	*p* value^a^
Primary care visits	5.34 ± 5.11 (4.00, 5.67)	5.84 ± 5.72 (4.50, 7.00)	*p* < 0.001	0.50 ± 1.93 (0.00, 0.14)	0.41 ± 1.95 (0.00, 0.00)	*p* < 0.001
Specialist care visits (not requiring referral from a primary care physician)^b^	1.89 ± 2.59 (1.00, 2.16)	2.02 ± 3.19 (1.00, 2.50)	*p* < 0.001	0.41 ± 1.19 (0.00, 0.28)	0.38 ± 1.30 (0.00, 0.00)	*p* < 0.001
Specialist care visits (requiring referral from a primary care physician)^c^	0.21 ± 0.62 (0.00, 0.14)	0.20 ± 0.68 (0.00, 0.00)	*p* < 0.001	0.05 ± 0.26 (0.00, 0.00)	0.05 ± 0.31 (0.00, 0.00)	*p* < 0.001
MRI and CT scans	0.05 ± 0.19 (0.00, 0.00)	0.11 ± 0.38 (0.00, 0.00)	*p* < 0.001	0.03 ± 0.17 (0.00, 0.00)	0.02 ± 0.21 (0.00, 0.00)	*p* < 0.001
Emergency room visits	0.13 ± 0.29 (0.00, 0.14)	0.54 ± 2.01 (0.00, 0.5)	*p* < 0.001	0.40 ± 2.11 (0.00, 0.28)	0.16 ± 1.85 (0.00, 0.00)	*p* < 0.001
Number of hospital admissions	0.00 ± 0.00 (0.00, 0.00)	0.05 ± 0.31 (0.00, 0.00)	*p* < 0.001	0.09 ± 0.40 (0.00, 0.00)	0.03 ± 0.32 (0.00, 0.00)	*p* < 0.001
Hospitalization days	0.00 ± 0.00 (0.00, 0.00)	0.16 ± 1.52 (0.00, 0.00)	*p* < 0.001	0.28 ± 1.91 (0.00, 0.00)	0.11 ± 2.02 (0.00, 0.00)	*p* < 0.001
Elective surgeries	0.00 ± 0.06 (0.00, 0.00)	0.02 ± 0.13 (0.00, 0.00)	*p* < 0.001	0.05 ± 0.19 (0.00, 0.00)	0.02 ± 0.15 (0.00, 0.00)	*p* < 0.001

Values are average ± SD (median, IQR).

^a^Mann–Whitney U test.

^b^Ophthalmology, orthopedics, gynecology, general surgery, dermatology, otolaryngology.

^c^Gastroenterology and cardiology.

Table 4 presents the exponentiated coefficients of the core independent variables in the eight multivariable models. Parts a of the Table present services that, as hypothesized, increased following the hospital market entry. Parts b and c of Table 4 present unexpected change in HCU following this market entry. Specifically, part b presents services that did not change following the market entry and part c present services that decreased following it.

**Table 4:** The difference-in-difference exponentiated coefficient^a^ (in bold), by type of service.

Type of service	Variable	IRR	95% CI	*p* value
A. Services’ utilization that increased post-market entry				
Specialist care visits (not requiring referral from a primary care physician)^b^	Post- (vs. pre-market entry)	0.98	0.97–0.99	*p* = 0.004
	Region (Ashdod vs. Netanya)	0.96	0.95–0.97	*p* < 0.001
	Post * Region^**c**^	**1.04**	**1.03–1.06**	***p* < 0.001**
MRI and CT scans	Post- (vs. pre-market entry)	1.06	1.03–1.10	*p* < 0.001
	Region (Ashdod vs. Netanya)	1.21	1.17–1.24	*p* < 0.001
	Post * Region^**c**^	**1.04**	**1.01–1.08**	***p* = 0.022**
Emergency room visits	Post- (vs. pre-market entry)	0.88	0.86–0.91	*p* < 0.001
	Region (Ashdod vs. Netanya)	1.22	1.19–1.25	*p* < 0.001
	Post * Region^**c**^	**1.33**	**1.29–1.38**	***p* < 0.001**
B. Services’ utilization that did not change post-market entry				
Number of hospital admissions	Post- (vs. pre-market entry)	1.09	1.02–1.16	*p* = 0.007
	Region (Ashdod vs. Netanya)	0.77	0.74–0.81	*p* < 0.001
	Post * Region^**c**^	**1.05**	**0.97–1.14**	***p* = 0.250**
Total hospitalization days	Post- (vs. pre-market entry)	1.13	1.09–1.18	*p* < 0.001
	Region (Ashdod vs. Netanya)	0.76	0.73–0.78	*p* < 0.001
	Post * Region^**c**^	**0.99**	**0.94–1.04**	***p* = 0.668**
C. Services’ utilization that decreased post-market entry				
Primary care visits	Post- (vs. pre-market entry)	1.05	1.04–1.06	*p* < 0.001
	Region (Ashdod vs. Netanya)	1.00	1.00–1.01	*p* = 0.303
	Post * Region^**c**^	**0.99**	**0.98–1.00**	***p* = 0.002**
Specialist care visits (requiring referral from a primary care physician)^d^	Post- (vs. pre-market entry)	0.90	0.88–0.92	*p* < 0.001
	Region (Ashdod vs. Netanya)	0.73	0.71–0.74	*p* < 0.001
	Post * Region^**c**^	**0.89**	**0.86–0.91**	***p* < 0.001**
Elective surgeries	Post- (vs. pre-market entry)	1.39	1.34–1.45	*p* < 0.001
	Region (Ashdod vs. Netanya)	1.24	1.21–1.27	*p* < 0.001
	Post * Region^**c**^	**0.58**	**0.55–0.60**	***p* < 0.001**

^a^All models were adjusted to age, sex, comorbidity burden, supplementary health insurance coverage, and SES.

^b^Ophthalmology, orthopedics, gynecology, general surgery, dermatology, otolaryngology.

^c^An interaction between region [Ashdod (study group) vs. Netanya (control group)] and post-market entry (vs. pre-market entry). This interaction term reflects the unique change in healthcare utilization of Ashdod region’s enrollees compared to Netanya region’s enrollees after the market entry.

^d^Gastroenterology and cardiology.

As depicted in Table 4A, and as hypothesized, after the market entry of Assuta Ashdod and compared to the control group, there was a 4% increase in the number of specialists visits not requiring referral (RR = 1.04, 95% CI 1.03–1.06, *p* < 0.001), a 4% increase in the number of MRI and CT scans (RR = 1.04, 95% CI 1.01–1.08, *p* = 0.022), and a 33% increase in emergency room visits (RR = 1.33, 95% CI 1.29–1.38, *p* < 0.001). Unexpectedly, as depicted in Table 4B, no changes in the number of hospital admissions (RR = 1.05, 95% CI 0.97–1.14, *p* = 0.250), and in total hospitalization days (RR = 0.99, 95% CI 0.94–1.04, *p* = 0.668) were observed following the market entry of Assuta Ashdod. Finally, and also unexpectedly, as depicted in Table 4C, there was a 1% decrease in the number of primary care visits (RR = 0.99, 95% CI 0.98–1.00, *p* = 0.002), a 11% decrease in specialists visits requiring a referral (RR = 0.89, 95% CI 0.86–0.91, *p* < 0.001), and a 42% decrease in the number of elective surgeries (RR = 0.58, 95% CI 0.55–0.60, *p* < 0.001).

## Discussion

From the perspective of the Israeli healthcare system, the market entry of Assuta Ashdod was a historic event that provided an opportunity to examine its effect on both community-based and hospital-based HCU. The study results reveal an increase in the utilization of emergency room visits, MRI and CT scans, and specialists visits not requiring a referral. However, unexpectedly, there was a decrease in the number of primary care visits, visits to gastroenterology and cardiology specialists (requiring a referral), and elective surgeries. In addition, the number of hospital admissions, and total hospitalization days did not change following the market entry.

The health system is characterized by asymmetric information between health providers and patients, which may lead to a supplier-induced demand^2^ and overuse of healthcare services. Many studies have examined this phenomenon in a wide range of health services^3–8^. Our results showing an increase in emergency room visits, MRI and CT scans, and specialist care visits correspond with this body of literature. The increase in the use of MRI and CT scans following the market entry is also similar to studies conducted in Ontario^25^ and Manaus^26^. Excess use of diagnostic procedures (such as MRI and CT scans) may be derived from defensive medicine, a desire to respond to patient demand requests, or a lack of communication between groups of doctors from different specialties such as general physicians, radiologists, and orthopedic specialists^25^. In this case, further study is required to investigate whether the MRI and CT scans are wisely utilized at Assuta Ashdod. Changes in payment methods to hospitals have been also linked to overuse. A study conducted in Swiss hospitals after significant reform in hospital payment methods towards the diagnosed related group found increased demand for MRI and CT scans in the ambulatory setting, whereas there was a decrease in the number performed during hospitalizations^17^. Thus, since hospitalizations in Assuta Ashdod are mostly paid per diem, these scanning tests may have been shifted to the outpatient setting where FFS model is applied.

With the opening of the emergency room at Assuta Ashdod in November 2017, the number of emergency room visits increase by 33% in the following two years, while there was only an average increase of 0.5% in the number of MHS enrollees in the city of Ashdod during the same period^27–29^. Similarly, a study from Australia demonstrated a higher increase in the number of emergency room visits following the opening of a new emergency department, compared to the rate of population growth during the study period. This increase was attributed to an unmet need^10^. Likewise, the opening of Assuta Ashdod provided a solution to scarce emergency services. Before the hospital was opened, clinically urgent cases were referred to hospitals that were located approximately 25 km away. The market entry of Assuta Ashdod was accompanied by a large health communication campaign, emphasizing the uniqueness of the hospital’s medical services including the 24/7 presence of physicians who specialized in emergency medicine, digital services, security considerations, innovative architecture, and patient privacy. We assume that all these amenities aroused curiosity among MHS enrollees. To avoid unnecessary use of these services, MHS developed an intervention program that included an expansion of community-based emergency medical services and strengthening the connection between hospital and community physicians. It is warranted to examine whether the new facility has reduced mortality rates, as well as other related health outcomes in the area.

Opening of outpatient clinics at Assuta Ashdod has increased availability and accessibility of these services for Ashdod area residents. Indeed, we observed increase in the utilization of specialist visits not requiring a referral, while the number of specialists visits requiring a referral (gastroenterology and cardiology) decreased after the market entry. We should note that both changes were relatively modest and may have been amplified due to the opposite trend that was observed in the control group (Netanya region). The cardiology department at Assuta Ashdod employs experts in various cardiology domains. Cardiologists working at the facility have developed direct communication with primary care physicians to create a treatment continuum for patients based on partnership and professional trust. In addition, some of them have also started working in community clinics to increase their exposure. However, our study findings may imply that MHS enrollees developed trust in and loyalty to their specialists—traits that are developed based on patient characteristics, expectations, availability, and duration of the physician–patient relationship. Patients mostly develop loyalty to the doctor and not to their office location^30, 31^. Patient satisfaction, trust, and loyalty also predict their recommendation of a specific doctor to others^32^. Because patient–physician relationships develop over time, a longer follow-up after the market entry may be required to identify whether the increase in accessibility of this care and integration of hospital specialists in community clinics will indeed increase visits to specialist care at Assuta Ashdod.

Another finding that emerged from our analyses is that there was no change in hospital admissions and total hospitalization days. This finding is not in line with Roemer’s Law^9^. In addition, it is not in agreement with the expected influence of the payment mechanism for hospitalization at Assuta Ashdod, which is per diem. This payment scheme incentivizes hospitals to increase hospitalization duration^19^—a gap that may stem from the unique model that was adopted by Assuta Ashdod of a therapeutic continuum using the Integration Unit, that consists of a multidisciplinary team of MHS staff that includes physicians, nurses, paramedical professionals, and administrative staff. The Integration Unit accompanies MHS patients and their families from the first day of admission, continuously communicating with their primary physicians, and plans patient discharge (e.g., rehabilitation care). This concept may shorten the hospitalization duration. It would be interesting to examine over time whether this trend continues compared to hospitals where no such unit exists, as well as to distinguish between new and repeated hospitalizations to assess the efficiency of such units.

Finally, our findings indicate a decrease in the number of elective surgeries after the market entry of Assuta Ashdod. This is contrary to the expected effect of supplier-induced demand and of payment arrangements^19^. It also differs from findings of a study conducted in the United States with the opening of ambulatory surgical centers that demonstrated an increase in the volume of activity that was not offset by a decrease in the volume of activity at the pre-existing medical centers^33^. Our observed decrease may stem from technical considerations, namely, changes that occurred after Assuta Ashdod was opened in the definition and coding of surgical procedures. Thus, further investigation is required into the type of procedures that showed a significant decrease.

Our analyses have several limitations. First, subjects were defined as MHS enrollees according to their eligibility at one point in time per research year; thus, it is possible that data on the HCU of MHS enrollees were estimated over a year, while in fact they were eligible during ≤ 12 months. However, since this definition was applied in the same way in both study groups and both pre- and post-market entry of Assuta Ashdod, we believe that its effect (if any) was common and applied to a similar extent across groups and throughout the study period; therefore, it did not affect the study findings. Second, data about the quality and availability of other healthcare services (e.g., wards in exiting medical centers and physicians) was not available, thus, the multivariable models were not adjusted to these variables albeit may affect both study and control groups differently. Third, our analyses refer to MHS enrollees of Ashdod region, thus, ignore the influence of the market entry of Assuta Ashdod on localities that are not included in this region. However, we believe that since we analyzed the major region that may have been affected by this market entry, our results are valid. Finally, the study examined data on HCU only two years after the market entry of Assuta Ashdod; therefore, it did not address the long-term effects of a new provider entering the healthcare system.

## Conclusion

This is the first study that examined how the market entry of Assuta Ashdod influenced both community- and hospital-based HCU. Our findings may be used as a model to characterize the inter-relationship between the hospital and community care. This market entry led to a shift in the geographical distribution of HCU from suppliers distant from the new provider to suppliers in proximity to this provider. But this was not necessarily translated to an increase in utilization of all inpatient and outpatient healthcare services in the short term. It is possible that the unique model of maintaining the continuity of care that was adopted by Assuta Ashdod and patients’ loyalty also led to the unique and unexpected patterns in HCU. Further study is required to examine long-term patterns of HCU following this market entry subject to expected changes in payment schemes, as well as the associated influence of this market entry on health outcomes.

## Data Availability

The data that support the findings of this study contains potentially identifiable patients’ information. Thus, following the requirements of MHS Internal Review Board–Helsinki Committee, the data are not publicly available. Data requests may be sent to the corresponding author (TST).

## Abbreviations

CT: Computerized tomography
DRG: Diagnosed-related group
FFS: Fee-for-service
GEE: Generalized estimating equations
HCU: Healthcare utilization
HMO: Health maintenance organization
MHS: Maccabi Healthcare Services
MRI: Magnetic resonance imaging
SES: Socioeconomic status

## References

[1] Arrow KJ (1963). Uncertainty and the welfare economics of medical care. Am. Econ. Rev..

[2] Sloan FA, Hsieh CR (2012). Health Economics.

[3] Delamater PL, Messina JP, Grady SC, WinklerPrins V, Shortridge AM (2013). Do more hospital beds lead to higher hospitalization rates? A spatial examination of Roemer’s law. PLoS ONE.

[4] Van Doorslaer E, Van Vliet R (1989). A built bed is a filled bed? An empirical reexamination. Soc. Sci. Med..

[5] Seyedin H, Afshari M, Isfahani P, Hasanzadeh E, Radinmanesh M (2021). The main factors of supplier-induced demand in health care: A qualitative study. J. Educ. Health Promot..

[6] Karimi S, Khorasani E, Keyvanara M, Afshari S (2015). Factors affecting physicians’ behaviors in induced demand for health services. Int. J. Educ. Psychol. Res..

[7] Yuda M (2013). Medical fee reforms, changes in medical supply densities, and supplier induces demand: Empirical evidence from Japan. Hitotsubashi J. Econ..

[8] Mohamadloo A, Zarein-Dolab S, Ramezankhani A, Salamzadeh J (2019). The main factors of induced demand for medicine prescription: A qualitative study. Iran. J. Pharm. Res..

[9] Shain M, Roemer MI (1959). Hospital costs relate to the supply of beds. Mod. Hosp..

[10] Crilly J, O’Dwyer J, Lind J, Tippett V, Thalib L, O’Dwyer M (2013). Impact of opening a new emergency department on health care service and patient outcomes: Analyses based on linking ambulance, emergency and hospital databases. Intern. Med. J..

[11] Alexander JA, Lee SYD, Griffith JR, Mick SS, Lin X, Banaszak-Holl J (1999). Do market-level hospital and physician resources affect small area variation in hospital use?. Med. Care Res. Rev..

[12] Crilly JL, Keijzers GB, Keijzers GB, Tippett VC, O'Dwyer JA, Wallis MC (2014). Expanding emergency department: A multisite study. Aust. Health Rev..

[13] OECD. Better ways to pay for health care, OECD health policy studies. https://www.oecd.org/publications/better-ways-to-pay-for-health-care-9789264258211-en.htm. Accessed 9 October 2021.

[14] Godson T, Forland F, Kristiansen IS, Sutton M, Leese B, Giuffrida A (2000). Capitation, salary, fee-for-service and mixed systems of payment: Effects on the behavior of primary care physicians. Cochrane Database Syst. Rev..

[15] Van Dijek CE, Van Den Berg B, Verheij RA, Spreeuwenberg P (2013). Moral hazard and supplier induced demand: Empirical evidence general practice. Health Econ..

[16] Palmer KS, Agoritsas T, Martin D, Scott T, Mulla SM, Miller AP (2014). Activity-based funding of hospitals and its impact on mortality, readmission, discharge destination, severity of illness, and volume of care: A systematic review and meta-analysis. PLoS ONE.

[17] Zabrodina V, Dusheiko M, Moschetti K (2020). A moneymaking scan: Dual reimbursement systems and supplier-induced demand for diagnostic imaging. Health Econ..

[18] Xiaoyu X, Ennan W, Qianni L, Piaopiao C, Tian W (2019). Does an economic incentive affect provider behavior? Evidence from a field experiment on different payment mechanisms. J. Med. Econ..

[19] Barber, S., Lorenzoni, L., Ong, P. Price setting and price regulation in health care. https://www.oecd.org/health/health-systems/OECD-WHO-Price-Setting-Summary-Report.pdf. Accessed 9 October 2021.

[20] Gaspar K, Portrait F, Van der Hijden E, Koolman X (2021). Global budget versus cost ceiling: A natural experiment in hospital payment reform in the Netherlands. Eur. J. Health Econ..

[21] Rosen B, Waitzberg R, Merkur S (2015). Israel: Health system review. Health Syst. Transit..

[22] The National Insurance Institute of Israel, Statistics by localities [Hebrew]. https://www.btl.gov.il/mediniyut/situation/statistics/btlstatistics.aspx. Accessed 1 May 2022.

[23] Ministry of Health Israel. Inequality in health and coping with it. 2017 [Hebrew]. https://www.health.gov.il/publicationsfiles/inequality-2017.pdf. Accessed 10 October 2021.

[24] Characterization and classification of statistical areas within municipalities and local councils by the socio-economic level of the population 2015 [Hebrew]. https://www.cbs.gov.il/en/mediarelease/pages/2019/characterization-and-classification-of-statistical-areas-within-municipalities-and-local-councils-by-the-socio-economic-lev.aspx. Accessed 10 October 2021.

[25] John JY, Levinso W, Laupacis A (2009). Attitudes of family physicians, specialists and radiologists about the use of computed tomography and magnetic resonance imaging in Ontario. Healthc. Policy.

[26] de Oliveira Andrade E, de Andrade EN, Gallo GH (2011). Case study of supply induced demand: The case of provision of imaging scans (computed tomography and magnetic resonance) at Unimed-Manaus. Rev. Assoc. Med. Bras..

[27] The National Insurance Institute of Israel. HMO membership. 2016 [Hebrew]. https://www.btl.gov.il/Publications/survey/Documents/seker289/seker_289.pdf. Accessed 9 October 2021.

[28] The National Insurance Institute of Israel. HMO membership 2018–2019. https://www.btl.gov.il/Publications/survey/Documents/seker317/seker_317.pdf. Accessed 9 October 2021.

[29] The National Insurance Institute of Israel. HMO membership 2017. https://www.btl.gov.il/Publications/survey/Documents/seker_303.pdf. Accessed 9 October 2021.

[30] Gérard L, François M, Chefdebien M, Saint-Lary O, Jami A (2016). The patient, the doctor, and the patient’s loyalty: A qualitative study in French general practice. Br. J. Gen. Pract..

[31] Roberge D, Beaulieu M, Haddad S, Lebeau R, Pineault R (2001). Loyalty to the regular care provider: Patients and physicians' views. Fam. Pract..

[32] Platonova E, Kennedy KN, Shewchuk R (2008). Understanding patient satisfaction, trust, and loyalty to primary care physicians. Med. Care Res. Rev..

[33] Courtemanchea C, Plotzke M (2010). Does competition from ambulatory surgical centers affect hospital surgical output?. J. Health Econ..

